# An Evaluation of Sensor Performance for Harmful Compounds by Using Photo-Induced Electron Transfer from Photosynthetic Membranes to Electrodes

**DOI:** 10.3390/s16040438

**Published:** 2016-03-25

**Authors:** Megumi Kasuno, Hiroki Kimura, Hisataka Yasutomo, Masaki Torimura, Daisuke Murakami, Yusuke Tsukatani, Satoshi Hanada, Takayuki Matsushita, Hiroaki Tao

**Affiliations:** 1 Department of Materials Chemistry, Faculty of Science and Technology, Ryukoku University, Otsu, Shiga 520-2194, Japan; c8w5k8@bma.biglobe.ne.jp (H.K.); rhodobacter.sphaeroides.hy@gmail.com (H.Y.); t-matsu@hcn.zaq.ne.jp (T.M.); 2 Research Institute for Environmental Management Technology, National Institute of Advanced Industrial Science and Technology (AIST), Tsukuba, Ibaraki 305-8569, Japan; daisuke.murakami0302@gmail.com (D.M.); hiro-tao@aist.go.jp (H.T.); 3 Earth-Life Science Institute, Tokyo Institute of Technology, Tokyo 152-8550, Japan; tsukatani@elsi.jp; 4 Institute for Bioproduction Research Institute, National Institute of Advanced Industrial Science and Technology (AIST), Tsukuba, Ibaraki 305-8566, Japan; s-hanada@aist.go.jp

**Keywords:** *Rhodobacter sphaeroides*, chromatophore, photo-induced electron transfer, Michaelis-Menten-type kinetics, carbon paste electrode, harmful compounds

## Abstract

Rapid, simple, and low-cost screening procedures are necessary for the detection of harmful compounds in the effluent that flows out of point sources such as industrial outfall. The present study investigated the effects on a novel sensor of harmful compounds such as KCN, phenol, and herbicides such as 3-(3,4-dichlorophenyl)-1,1-dimethylurea (DCMU), 2-chloro-4-ethylamino-6-isopropylamino-1,3,5-triazine (atrazine), and 2-*N*-tert-butyl-4-*N*-ethyl-6-methylsulfanyl-1,3,5-triazine-2,4-diamine (terbutryn). The sensor employed an electrode system that incorporated the photocurrent of intra-cytoplasmic membranes (so-called chromatophores) prepared from photosynthetic bacteria and linked using carbon paste electrodes. The amperometric curve (photocurrent-time curve) of photo-induced electron transfer from chromatophores of the purple photosynthetic bacterium *Rhodobacter sphaeroides* to the electrode via an exogenous electron acceptor was composed of two characteristic phases: an abrupt increase in current immediately after illumination (*I*_0_), and constant current over time (*I*_c_). Compared with other redox compounds, 2,5-dichloro-1,4-benzoquinone (DCBQ) was the most useful exogenous electron acceptor in this system. Photo-reduction of DCBQ exhibited Michaelis-Menten-like kinetics, and reduction rates were dependent on the amount of DCBQ and the photon flux intensity. The *I*_c_ decreased in the presence of KCN at concentrations over 0.05 μM (=μmol·dm^−3^). The *I*_0_ decreased following the addition of phenol at concentrations over 20 μM. The *I*_c_ was affected by terbutryn at concentrations over 10 μM. In contrast, DCMU and atrazine had no effect on either *I*_0_ or *I*_c_. The utility of this electrode system for the detection of harmful compounds is discussed.

## 1. Introduction

Whole effluent toxicity (WET) testing is an integral approach for detecting and addressing toxicity in surface waters and is recommended by the United States Environmental Protection Agency (US EPA). WET testing actually measures the adverse effects or toxicity to a population of aquatic organisms caused by exposure to an effluent that flows out of point sources such as industrial outfall. WET testing is used to assess and regulate the comprehensive effects of all constituents of the effluent, in contrast to conventional methods, which typically estimate the toxicity of single constituents [1,2,3]. The duration of the test may range from as short as 40 min to as long as seven days, depending on the organisms used and whether acute or chronic effects are of interest. Practical monitoring programs should involve rapid, simple, and low-cost screening procedures for the detection of harmful compounds in aquatic and soil environments.

Photosynthetic electron transfer reactions in purple photosynthetic bacteria can be affected by harmful compounds in wastewater effluent. For example, herbicides inhibit electron transfer in type-II reaction centers by substituting for the secondary electron acceptor quinone (Q_B_) in the reaction center [4,5,6,7]. *Rhodobacter sphaeroides* is a representative of purple photosynthetic bacteria, and the photocurrent generated in this organism’s reaction center has been extensively investigated as a potential biosensor for the classes of herbicides that inhibit type-II reaction centers. Several studies have shown that photocurrent generation in *R. sphaeroides* is inhibited by typical herbicides such as 2-chloro-4-ethylamino-6-isopropylamino-1,3,5-triazine (atrazine) and 2-*N*-tert-butyl-4-*N*-ethyl-6-methylsulfanyl-1,3,5-triazine-2,4-diamine (terbutryn) [8,9,10]. Use of the *R. sphaeroides* photocurrent as a biosensor is based on the fact that various pollutants and toxic compounds found in water are known to inhibit photosynthetic electron transport in this organism. As not only herbicides but also cyanide ion (CN^−^) and heavy metals inhibit the photosynthetic reaction [11,12,13,14,15], it is believed that photosynthetic bacteria would be useful indicators of harmful compounds in waste effluents as well as for use in WET testing.

Most of the purple photosynthetic bacteria, including *R. sphaeroides*, are easy to cultivate, and characteristically, harbor intra-cytoplasmic photosynthetic membranes, so-called “chromatophores” [16,17]. We previously developed a system that used an exogenous mediator and a rotating disk electrode to measure photo-induced electron transfer from chromatophores or from cells of *R. sphaeroides* [18]. The photo-induced electron transfer from chromatophores or intact cells to the electrode was found to follow Michaelis-Menten-type kinetics. The Michaelis constants and maximum photoinduced electron transfer reaction rates were evaluated to guide further system design. Chromatophore vesicles were found to be more stable than intact cells and retain the ability to mediate the photocurrent for at least one month. Additionally, terbutryn was shown to inhibit the photocurrent resulting from electron transfer from the chromatophores to the electrode. Though rotating disk electrodes are useful for kinetic investigations of photo-induced electron transfer, such electrodes are difficult to use as the sensor for detecting toxic substances in the field. On the other hand, carbon paste electrodes (CPEs) are more easily adopted for use in the field, and can be modified with chromatophore vesicles and the addition of an exogenous mediator [19,20,21]. Therefore, CPEs are preferable for the sensing of toxic substances in the field.

In the present work, we established a CPE system for measuring photo-induced electron transfer from *R. sphaeroides* chromatophore vesicles to the exogenous mediator. The effect of harmful compounds such as CN^−^, phenol, and typical agricultural chemicals on electron transfer in this system also was investigated, permitting evaluation of the sensor performance of the CPE system incorporating *R. sphaeroides* chromatophores. The mechanism of electron transfer reaction from chromatophore to CPE via the exogenous mediator is the same as that indicated in Figure in reference [21], a study that investigated the photo-induced electron transfer to CPE from the membrane-localized type-II reaction center of spinach.

## 2. Materials and Methods

### 2.1. Preparation of Chromatophore Vesicles and Reagents

The purple photosynthetic bacterium used in this study, *R. sphaeroides* strain NBRC 12203, was obtained from the culture collection department of the National Institute of Technology and Evaluation (Tokyo, Japan). Bacterial cultivation and chromatophore vesicle preparation were done according to previously described procedures [18]. The concentration of chromatophore vesicles was defined as the amount of bacteriochlorophyll (BChl) *a* pigments. Concentrations of BChl *a* ([BChl]) in the *R. sphaeroides* chromatophore suspension were determined by measuring the absorbance at 773 nm using an extinction coefficient of 75 mM^−1^·cm^−1^ (M = mol·dm^−3^) [22].

2,5-Dichloro-1,4-benzoquinone (DCBQ; Wako Pure Chemical Industries Ltd., Osaka, Japan ), 2,5-dihydroxy-1,4-benzoquinone (DHBQ; Sigma-Aldrich Co. LLC, Tokyo, japan), 1,4-benzoquinone (BQ; Wako Pure Chemical Industries Ltd., Osaka, Japan), and potassium hexacyanoferrate(III) (K3[Fe(CN)6]; Wako Pure Chemical Industries Ltd., Osaka, Japan) were tested as exogenous mediators. Potassium cyanide (KCN; Wako Pure Chemical Industries Ltd., Osaka, Japan), phenol (Wako Pure Chemical Industries Ltd., Osaka, Japan), 3-(3,4-dichlorophenyl)-1,1-dimethylurea (DCMU; Sigma-Aldrich Co. LLC, Tokyo, japan), atrazine (Sigma-Aldrich Co. LLC, Tokyo, Japan), and terbutryn (Sigma-Aldrich Co. LLC, Tokyo, japan) were used as inhibitors. The pH of the aqueous solution was adjusted to 8.0 with phosphate buffer (50 mM of K_2_HPO_4_/KH_2_PO_4_).

### 2.2. Fabrication of R. sphaeroides Chromatophore-Entrapped and Mediator-Embedded CPEs

Mediator-modified CPEs were prepared by reference to a previously described method [19]. A weighed amount of the mediator was mixed with a weighed amount of a carbon paste (BAS Co. Ltd., Osaka, Japan). The mixture of mediator and carbon paste was packed into a hole of the electrode. The surface was smoothed using powder paper. The geometric surface area of the electrode was 0.07 cm^2^. The amount of mediator was expressed as the fraction of mediator in the carbon paste mixture, (mediator)_m_% (*w*/*w*) [21]. Aliquots of *R. sphaeroides* chromatophore suspension were placed dropwise onto the surface of the CPE. After the solvent evaporated, the electrode surface was covered with a 20-μm-thick dialysis membrane (No. 20/30; Union Carbide Co., Houston, TX, USA), which was fixed in place with a nylon net. The amount of BChl *a* entrapped at the CPE was estimated based on [BChl] and the volume of the chromatophore suspension dropped onto the CPE. Electrodes prepared in this way (abbreviated as *R. sph*.-mediator-CPEs) were maintained in 50 mM of phosphate buffer (pH 8.0) at 25 ± 1 °C in the dark and were used as working electrodes within 1 day of preparation. Based on the relationship between the amount of BChl *a* and the photo-induced current value, the amount of *R. sphaeroides* chromatophores corresponding to that of BChl *a* of 5 μmol was adopted in the present work, as described in detail below.

### 2.3. Electrochemical Measurements

All electrochemical measurements were carried out using a single-compartment cell containing a three-electrode configuration at 25 ± 1 °C under deaerated conditions created by passing argon gas through a 50 mM phosphate buffer solution (pH 8.0). A silver/silver chloride wire in saturated KCl (SSE) and a platinum wire served as the reference and counter electrodes, respectively.

Amperometric measurements were carried out by applying a potential of 0.50 V *vs.* SSE (the diffusion-controlled region for oxidation of the reduced mediator) to the *R. sph*.-mediator-CPE using a BAS CV27 potentiostat (BAS Co. Ltd., Osaka, Japan) in connection with a model F-35Fm X-Y recorder (Riken Denshi Co., Ltd., Tokyo, Japan). Here, the mediator oxidation by the diffusion control was observed in cyclic voltammograms recorded with the *R. sph*.-mediator-CPE at around 0.5 V when the (mediator)_m_ was in the range between 0.05% and 1.0%.

Light at wavelengths longer than 660 nm was supplied from the bottom of the cell by a light source (LG-PS2, Olympus Optical Co., Ltd., Tokyo, Japan) through a R66 glass filter (HOYA Candeo Optronics, Saitama, Japan), and the light intensity was measured using a light meter (PM10; Coherent, Inc., Santa Clara, CA, USA) [18].

The effect of potentially harmful test compounds on the photocurrent was examined 10 min after the addition of an aliquot of the stock solution containing the test compound. Time was sufficient to allow the reaction between the test compound and the chromatophore to reach equilibrium. The degree of the test compound effect on the photocurrent was evaluated at each concentration of the test compound. Five *R. sph*.-mediator-CPEs were fabricated and used for the evaluation of the test compound effect at each concentration. The precision was calculated from results of these five measurements.

## 3. Results and Discussion

### 3.1. Characteristics of Photo-Induced Electron Transfer from R. sphaeroides Chromatophores to CPEs through Exogenous Electron Acceptors

**Figure 1:**
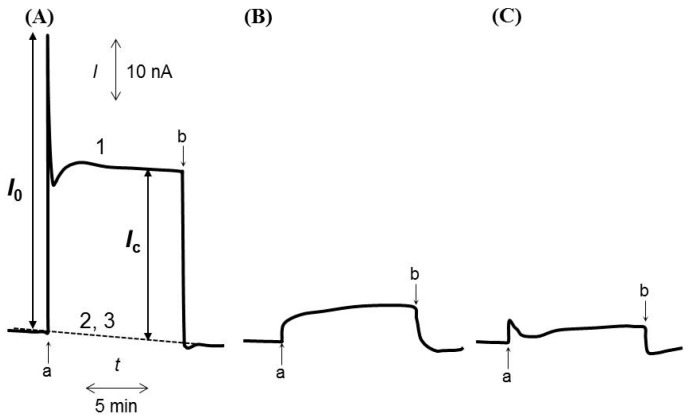
(**A**) Time-course of the amperometric response measured using a *R. sph.* -DCBQ (0.5%)-CPE (curve 1), *R. sph.*-CPE (curve 2), or DCBQ (0.5%)-CPE (curve 3) in pH 8.0 buffer. (**B**,**C**) Time-courses of the amperometric response measured under the same conditions as (**A**), but with DHBQ or BQ used as the mediator. Light illumination was applied between points “a” and “b.”

Curve 1 in Figure 1A shows the amperometric response measured using an *R. sph.*-DCBQ-CPE, followed by light illumination between points “a” and “b.” The amount of BChl *a* and (DCBQ)_m_ were 5 μmol and 0.5%, respectively. The photon flux intensity ([P]) was 500 μmol·m^−2^·s^−1^. An abrupt increase in the positive current (*I*_0_) was observed immediately after illumination (point a), followed by a gradual decrease to a constant current value (*I*_c_) by 3 min, with the positive current decreasing to the background level once the light was turned off (point b). This photocurrent was not observed in the absence of *R. sphaeroides* chromatophores (curve 2) or DCBQ (curve 3) upon illumination. The photocurrent is anodic, meaning that electrons are transferred predominantly from the *R. sphaeroides* chromatophores to the CPE via interaction with the mediator. Here, DCBQ, photo-reduced by the *R. sphaeroides* chromatophore photosynthetic electron transport system, was re-oxidized at the CPE. The *I*_0_ and *I*_c_ values were obtained through repeated measurements (*n* = 5), with an accuracy of ±2 and ±3%, respectively, using the same *R. sph.*-DCBQ-CPE, and with an accuracy of ±9 and ±7%, respectively, with different *R. sph.*-DCBQ-CPEs prepared using the same procedure. The *I*_0_ and *I*_c_ reflect the photo-induced electron transfer rate immediately after illumination and in the equilibrium state, respectively. When the rotating disk electrode was used, an initial abrupt increase in the positive current was considered to be attributable to the adsorption of *R. sphaeroides* chromatophores to the electrode [18]. The *I*_0_ in Figure 1A may include an element of *R. sphaeroides* chromatophore adsorption at the surface of the CPE.

Experiments similar to that illustrated in Figure 1A were performed using DHBQ (Figure 1B) or BQ (Figure 1C) instead of DCBQ. The formal potentials (*E*°′) of DCBQ, DHBQ, and BQ are reportedly 0.302, 0.380, and 0.099 V *versus* normal hydrogen electrode (NHE), respectively [23]. As with DCBQ, DHBQ functions as an exogenous electron acceptor for the *R. sphaeroides* chromatophore photosynthetic electron transport system. However, the rate of electron transfer from the chromatophore to DHBQ was found to be significantly slower than the rate of transfer to DCBQ. It is possible that reduced DHBQ donates electrons to the photosynthetic electron transport system in *R. sphaeroides* chromatophores, as has been suggested in the literature [24]. When BQ was used as the exogenous mediator, a photocurrent (lower than that obtained with DCBQ) was observed. This result was deemed reasonable (in energetic terms) based on the *E*°′ values of DCBQ and BQ [23]. Energetically, it is also possible that [Fe(CN)_6_]^3−^ can accept an electron from the *R. sphaeroides* chromatophore because the *E*°′ value of [Fe(CN)_6_]^3−^ is larger (0.443 V *vs.* NHE) than that of DCBQ [23]. However, photoreduction of [Fe(CN)_6_]^3−^ by the chromatophore was not observed. In this context, Izawa has pointed out that the accessibility of the type-II reaction center reduction sites (Q_A_ and Q_B_) to highly polar oxidants such as [Fe(CN)_6_]^3−^ seems to be very limited [24]. Matsue *et al.* have reported that the permeability of the membrane for the passage of [Fe(CN)_6_]^3−^ is small, but *p*-hydroquinone (QH_2_) can permeate the membrane more easily than [Fe(CN)_6_]^3−^ can [25]. BQ has been found to rapidly permeate the membrane, as does QH_2_ [25]. Lemaître *et al.* have suggested that several additional parameters (such as the kinetics of membrane transit) should be taken into account to explain the ability of quinones to access the photosynthetic electrons [26]. Since [Fe(CN)_6_]^3−^ is more hydrophilic than DCBQ, the difference in the photocurrent obtained with [Fe(CN)_6_]^3−^ and DCBQ would be attributed to limited membrane permeability of the [Fe(CN)_6_]^3−^.

The electron transfer from *R. sphaeroides* chromatophores to the exogenous mediator is believed to be governed not only by the redox potential of the exogenous mediator but also by the membrane penetrability of the exogenous mediators. The results of Figure 1 also indicate that, compared with other redox compounds, DCBQ is the most appropriate exogenous mediator for this system, as observed previously in other systems [24,26].

### 3.2. Effects on the Photocurrent of Varying R. sphaeroides Chromatophore Amount, DCBQ Fraction, and Light Intensity

As it is impossible to precisely estimate the amount of chromatophore vesicles in this system, we defined this value based on the amount of BChl *a*, as described in Section 2.1 above. The *I*_c_ was found to be proportional to the amount of BChl *a* for amounts under 4 μmol, according to Equation (1):

(1) $$I_{c}=nFAk\left[\text{BChl}\right]d$$

where *n*, *F*, *A*, and *d* represent the number of electrons, the Faraday constant, the surface area of the electrode, and the thickness of the immobilized chromatophore membrane layer, respectively. *k* represents the constant incorporating the correction factors for the diffusional effect of the mediator and the rate constants for the reactions [21]. As the amount of immobilized chromatophores increased, the positive current increased non-linearly, indicating that the photo-induced electron transfer reaction is not controlled by the amount of immobilized chromatophores. The photons may be diminished in the vicinity of the electrode surface by the presence of the chromatophore membrane. Based on the relationship between the amount of BChl *a* and *I*_c_, the subsequent experiments were carried out with the amount of BChl *a* set at 5 μmol.

**Figure 2:**
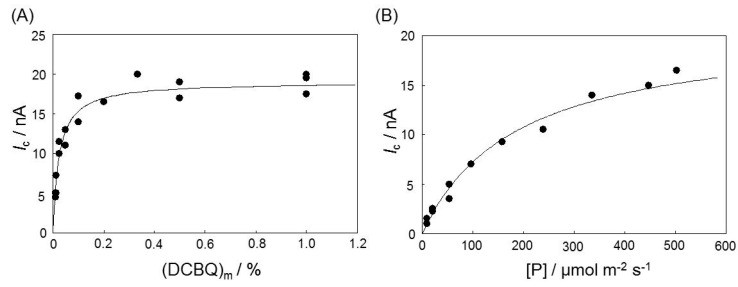
Dependence of the amount of DCBQ embedded in the CPE (**A**) or the photon flux intensity (**B**) on the photocurrent (*I*_c_). Lines represent the regression curves as determined using Equation (2).

It has been reported that quinone compounds, when mixed with CPEs with an immobilized enzyme layer on the electrode surface, dissolve (to some extent) in the enzyme layer, such that the concentration of quinone in the enzyme layer is proportional to the amount of quinone mixed with the CPEs [20,21]. Therefore, (DCBQ)_m_ is expected to be proportional to the concentration of DCBQ in the membrane layer of the immobilized *R. sphaeroides* chromatophores ([DCBQ]). Figure 2A shows a plot of *I*_c_ as a function of (DCBQ)_m_. The value of *I*_c_ increases with (DCBQ)_m_ until *I*_c_ approaches the maximum current value (*I*_max_). The redox reactions appear to follow Michaelis-Menten-like kinetics with respect to (DCBQ)_m_. As in previous studies [18,21], the plot in Figure 2A was analyzed according to Equation (2):

(2) $$I_{c}=I_{\text{max}}/\left[1+\left(K_{M}/{\left(DCBQ\right)}_{m}\right)\right]$$

where *K*_M_ represents the apparent Michaelis constant for (DCBQ)_m_. The values of *I*_max_ and *K*_M_ were determined as 18.7 nA and 0.024%, respectively, by applying nonlinear curve fitting based on regression analysis of Equation (2). Here, the [DCBQ] value was estimated to be *ca.* 4 × 10^−5^ M at 0.5% of (DCBQ)_m_ from the voltammogram observed at the *R. sph.*-DCBQ-CPE by the method described by Ikeda *et al.* [20]. Therefore, the *K*_M_ value obtained at the CPE is expected to be *ca.* 2 × 10^−6^ M, which is relatively close to the *K*_M_ value previously obtained using the rotating disk electrode (7.8 × 10^−6^ M) [18]. In the photocurrent system between 2,6-dimethylbenzoquinone (DMBQ) and the type-II reaction center of spinach, the *K*_M_ value was evaluated to be 7.0 × 10^−5^ M [21], which was larger than that obtained in the present work. This indicates that the affinity of the exogenous mediator for the *R. sphaeroides* is higher than that for spinach. However, experimental conditions were different in our experiments, so it is difficult to compare the *I*_max_ values directly.

*I*_c_ also was plotted as a function of [P], as shown in Figure 2B, and this relationship also appeared to follow Michaelis-Menten-like kinetics with respect to [P]. The (DCBQ)_m_ was 0.5%. By substituting (DCBQ)_m_ and *K*_M_ for [P] and the apparent Michaelis constant for [P] (*K*_L_), respectively, in Equation (2), the *I*_max_ and *K*_L_ values were determined as 19.2 nA and 180.3 μmol·m^−2^·s^−1^, respectively. The curves in Figure 2A,B illustrate the results of the regression analyses. Based on the results described above, all experiments in the present work were carried out at a (DCBQ)_m_ of 0.5% and [P] of 500 μmol·m^−2^·s^−1^.

### 3.3. Effect of CN^−^, Phenol or Terbutryn on Electron Transfer from R. sphaeroides Chromatophores to CPE via DCBQ

**Figure 3:**
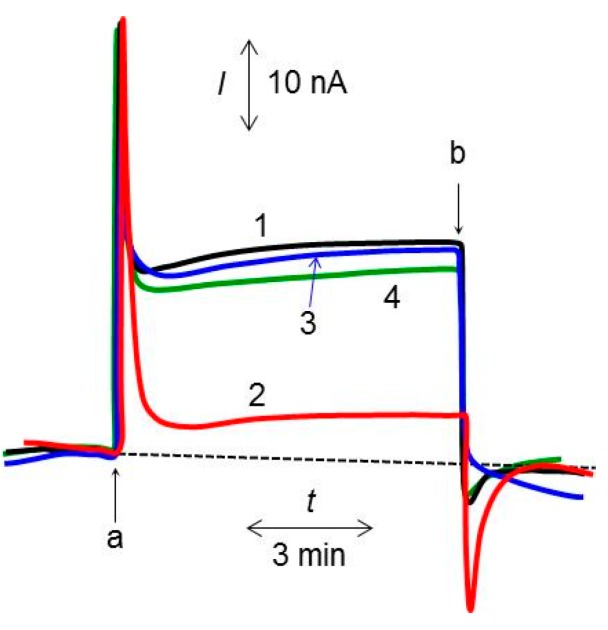
Time-course of the amperometric response measured using a *R. sph.*-DCBQ-CPE in the absence (curve 1) or presence of 5 μM of KCN (curve 2), 100 μM of phenol (curve 3) and 20 μM of terbutryn (curve 4). Light illumination was applied between points “a” and “b.”

Figure 3 shows the amperometric response measured using *R. sph.*-DCBQ-CPEs in a pH-8.0 buffer solution in the absence (curve 1) or presence of 5 μM of KCN (curve 2), 100 μM of phenol (curve 3) or 20 μM of terbutryn (curve 4) under light illumination between points “a” and “b.” Although the *I*_0_ value did not change, *I*_c_ decreased following the addition of KCN or terbutryn. In contrast to the results obtained with CN^−^ or terbutryn, the *I*_0_ decreased following the addition of phenol. The magnitude of *I*_c_ in curve 2 remained unchanged even after the *R. sph.*-DCBQ-CPE was thoroughly washed with distilled water and then transferred to a cell containing fresh buffer without CN^−^ or terbutryn. This observation indicates that, once “poisoned” with CN^−^ or terbutryn, *R. sph.*-DCBQ-CPEs do not recover their original activity and are therefore no longer capable of producing *I*_c_ at the original magnitude. The *I*_0_ value returned to near the original value after the *R. sph.*-DCBQ-CPEs were rinsed in the same way and re-used in fresh buffer without phenol. This result indicates that *R. sph.*-DCBQ-CPEs recover their original activity with respect to monitoring for phenol by washing of the electrode; that is, the inhibitory effect of phenol on the activity of *R. sphaeroides* chromatophores is reversible. The similar decrease of *I*_0_ was observed again by immersing washed *R. sph.*-DCBQ-CPE to phenol solution.

The ability of CN^−^ to inhibit photo-induced electron transfer is thought to be due to the strong binding of CN^−^ to cytochromes, such as cytochrome *c*, resulting in a shift in the redox potential of the Q_i_ site [27]. Ramasamy *et al.* have noted that KCN decreased the photocurrent, as measured using the cyanobacterium *Nostoc* sp*.* and BQ as the exogenous mediator; the effect was postulated to occur via inhibition of the transfer of electrons from cytochromes to PSI [28]. Terbutryn is reportedly more effective than other triazine-class inhibitors for interfering with electron transport from Q_A_ to Q_B_ in purple bacteria [4,6]. Therefore, the effect on *I*_c_ is considered to reflect the re-oxidation of DCBQ that has been reduced not only by cytochromes in the Q_i_ but also by Q_B_ in the electron transport chain. Phenol and its derivatives are thought to specifically inhibit the Q_i_ site [29]. Phenol also reportedly acts as an uncoupler of phosphorylation or an inhibitor of electron transfer in photosystems, depending on the concentration [30]. The *I*_0_ appeared to be due primarily to the re-oxidation of DCBQ that had been reduced by cytochrome *b_L_* or ubiquinone in the Q_i_ site. However, antimycin A, which interrupts electron transfer from cytochrome *b_L_* to ubiquinone in the Q_i_ site [31], had no effect on *I*_0_ or *I*_c_. Therefore, phenol may impair the proton gradient coupled with ATP synthesis, resulting in the decrease in *I*_0_ observed in the present study. The inhibitory effect of phenol was reversible, suggesting that *I*_c_ may not have been significantly affected by phenol. The current trough of curve 1 in Figure 1A is presumed to be due to a mixed or competing process, since the mechanisms for the observation of *I*_c_ and *I*_0_ may be different.

**Figure 4:**
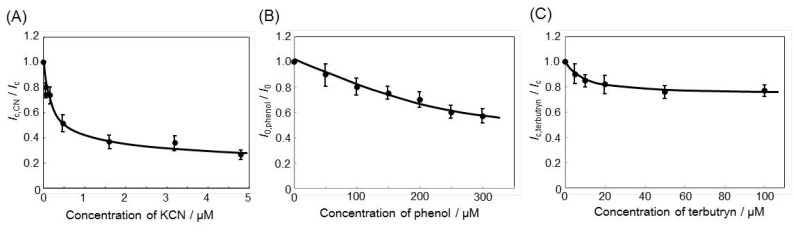
Effect of KCN (**A**) phenol; (**B**) or terbutryn; (**C**) concentration on the photocurrent (*I*_c_).

The ratio of the *I*_c_ value obtained with CN^−^ to that obtained without CN^−^, *I*_c,CN_/*I*_c_, was plotted as a function of the concentration of CN^−^ added to the buffer (Figure 4A). The *I*_c,CN_/*I*_c_ decreased to *ca.* 0.8 at a CN^−^ concentration of 0.01 μM and to *ca.* 0.3 at CN^−^ concentrations over 1.5 μM. With respect to limits for cyanide in drinking water, the World Health Organization suggests a guideline value of 0.07 mg·L^−1^ [32], which is detectable using the *R. sph.*-DCBQ-CPE system. Given that the detection limits of CN^−^ biosensors have been reported to be in the range 0.01–10 μM [33], the *R. sph.*-DCBQ-CPE system could be used for environmental detection of CN^−^.

The ratio of the *I*_0_ value obtained with phenol to that obtained without phenol, *I*_0,phenol_/*I*_0_, was plotted as a function of the concentration of phenol (Figure 4B). The *I*_0,phenol_ decreased gradually with increasing phenol concentration. At phenol concentrations of 100 and 300 μM, the resulting *I*_0,phenol_/*I*_0_ values were *ca.* 0.8 and 0.6, respectively. The detection limit of existing phenol biosensors is reportedly in the low micromolar range [34]; therefore, the sensitivity of the *R. sph.*-DCBQ-CPE system is not superior to that of previously reported systems. Although the *R. sph.*-DCBQ-CPE system is suitable for the detection of phenol at concentrations over 50 μM, other purple bacteria should be examined for more sensitive detection of this compound.

Triazine-type herbicides block electron transfer from Q_A_ to Q_B_ in the electron transport chain of purple bacteria, whereas urea- and phenol-type herbicides do not [4,35]. Neither *I*_0_ nor *I*_c_ was affected by the addition of DCMU or atrazine to the *R. sph.*-DCBQ-CPE system, even at a DCMU or atrazine concentration of 500 μM. In contrast, terbutryn affected *I*_c_ only at concentrations over 10 μM. The ratio of the *I*_c_ value obtained with terbutryn to that obtained without terbutryn, *I*_c,terbutryn_/*I*_c_, was *ca.* 0.8 at terbutryn concentrations over 20 μM. In a previous study, the activity of *R. sphaeroides* chromatophores at terbutryn concentrations over 50 μM was found to be *ca.* 80% that of the original value [18]. Jones *et al.* reported that the photocurrent was inhibited by atrazine and terbutryn and that the values of detection limits were 49 nM for atrazine and 8.3 nM for terbutryn [8]. Nagy *et al.* reported inhibition constants for terbutryn and atrazine of 1.3 and 63 μM, respectively, when assayed against the photosynthetic reaction center isolated from *R. sphaeroides* [36]. Nagy *et al.* further demonstrated that the three parameters (herbicide molecular structure, interaction between herbicide and quinone binding site, and protein environment) depended on the magnitude of the inhibitory effect of herbicides. Schmid *et al.* monitored the time-resolved absorption changes of the photoreactive center, and determined a detection limit of 0.04 μM for terbutryn [5]. In contrast to the results of those previous studies, the *R. sph.*-DCBQ-CPE system of the present study was not very sensitive to herbicides. This difference in herbicide sensitivity presumably reflects our use of *R. sphaeroides* chromatophore membranes (rather than isolated reaction centers), as well as differences in experimental conditions (e.g., the specific exogenous mediator used). To improve the sensitivity of the *R. sph.*-DCBQ-CPE system to herbicides, it may be necessary to fabricate the electrode by a method that maintains the membrane orientation of the *R. sphaeroides* component.

## 4. Conclusions

The present study demonstrated the detection of photo-induced electron transfer from *R. sphaeroides* chromatophores to an electrode via a mediator using a *R. sph.*-mediator-CPE system. DCBQ was found to be useful for use as the exogenous mediator with the *R. sph.*-mediator-CPE system. Electron transfer in *R. sphaeroides* chromatophores was impacted by harmful compounds such as CN^−^, phenol, and terbutryn. The *R. sph.*-DCBQ-CPE system constituted a particularly sensitive CN^−^ sensor. In addition, given that the photocurrent of chromatophore vesicles is more stable than that of live cells, the system described herein represents an attractive technology for sensing harmful compounds in wastewater effluents as well as for use in a WET testing system. The sensitivity of the *R.sph*.-DCBQ-CPE system to phenol and herbicides would need to be improved by some orders of magnitude. In order to fabricate a sensor for detecting a wide variety of phenols and herbicides, it may be more effective to develop systems incorporating photosynthetic organisms other than purple bacteria and plants.
